# Report of the AAPS Guidance Forum on the FDA Draft Guidance for Industry: “Drug Products, Including Biological Products, that Contain Nanomaterials”

**DOI:** 10.1208/s12248-019-0329-7

**Published:** 2019-04-17

**Authors:** Jon S. B. de Vlieger, Daan J. A. Crommelin, Katherine Tyner, Daryl C. Drummond, Wenlei Jiang, Scott E. McNeil, Sesha Neervannan, Rachael M. Crist, Vinod P. Shah

**Affiliations:** 1 Foundation Lygature, Utrecht, The Netherlands; 2 Department of Pharmaceutics, Utrecht University, Utrecht, The Netherlands; 3 Center for Drug Evaluation and Research, Office of Pharmaceutical Quality, US Food and Drug Administration, Silver Spring, Maryland USA; 4 Merrimack Pharmaceuticals, Cambridge, Massachusetts USA; 5 Center for Drug Evaluation and Research, Office of Generic Drugs, Office of Research and Standards, US Food and Drug Administration, Silver Spring, Maryland USA; 6 Nanotechnology Characterization Laboratory, Cancer Research Technology Program, Frederick National Laboratory for Cancer Research, Frederick, Maryland USA; 7 Allergan Plc, Irvine, California USA; 8 VPS Consulting LLC, North Potomac, Maryland USA

## Abstract

To guide developers of innovative and generic drug products that contain nanomaterials, the U.S. Food and Drug Administration issued the draft guidance for industry titled: “Drug Products, Including Biological Products, that Contain Nanomaterials” in December 2017. During the AAPS Guidance Forum on September 11, 2018, participants from industry, academia, and regulatory bodies discussed this draft guidance in an open setting. Two questions raised by the AAPS membership were discussed in more detail: *what is the appropriate regulatory pathway for approval of drug products containing nanomaterials*, and *how to determine critical quality attributes (CQAs) for nanomaterials?* During the meeting, clarification was provided on how the new FDA center-led guidance relates to older, specific nanomaterial class, or specific product-related guidances. The lively discussions concluded with some clear observations and recommendations: (I) Important lessons can be learned from how CQAs were determined for, e.g., biologics. (II) Publication of ongoing scientific discussions on strategies and studies determining CQAs of drug products containing nanomaterials will significantly strengthen the science base on this topic. Furthermore, (III) alignment on a global level on how to address new questions regarding nanomedicine development protocols will add to efficient development and approval of these much needed candidate nanomedicines (innovative and generic). Public meetings such as the AAPS Guidance Forum may serve as the place to have these discussions.

## INTRODUCTION

In Spring 2018, AAPS circulated a survey among its members asking (i) which FDA guidance(s) the community would most benefit from discussing in a workshop setting and (ii) what challenges should be discussed related to the guidance. The outcome suggested two draft guidance documents: the FDA draft guidance “Drug Products, Including Biological Products, that Contain Nanomaterials” (the nanomaterial guidance), published in December 2017 (1), and the FDA draft guidance on “Assay Development and Validation for Immunogenicity Testing of Therapeutic Protein Products,” published in April 2016 (2). During two adjacent days, September 11–12, 2018, participants discussed the two draft guidance documents. Since the scope of the two guidance documents, as well as the composition of the audience, differed significantly, this report focuses only on the draft guidance on drug products containing nanomaterials. Discussions around the second draft guidance on immunogenicity testing are covered in a separate report.

In response to the AAPS survey, two specific questions came up related to the nanomaterial guidance: *what is the appropriate regulatory pathway for approval of drug products containing nanomaterials*, and *how to determine critical quality attributes for nanomaterials?* Discussing these two questions will provide developers of drug products containing nanomaterials important insights for their New Drug Applications (NDA) and Abbreviated New Drug Applications (ANDA) submissions. Hence, these topics, and others described below, were the subject of a lively debate among scientists from industry, academia, and regulatory authorities at the AAPS Guidance Forum workshop on September 11, 2018. This report summarizes the presentations and deliberations at the workshop.

## DEFINING THE FIELD

Following a brief introduction by Dr. Vinod Shah, the chair of the workshop, speakers from the FDA and industry presented their thoughts about the background and content of the recently published draft guidance which covers various elements of the regulatory recommendations for drug products containing nanomaterials. Dr. Katherine Tyner from FDA (CDER Nanotechnology Working Group Lead) kicked off the presentations by describing her personal views regarding the history and reasoning behind the guidance document before discussing parts of the text in more detail.

**Fig. 1:**
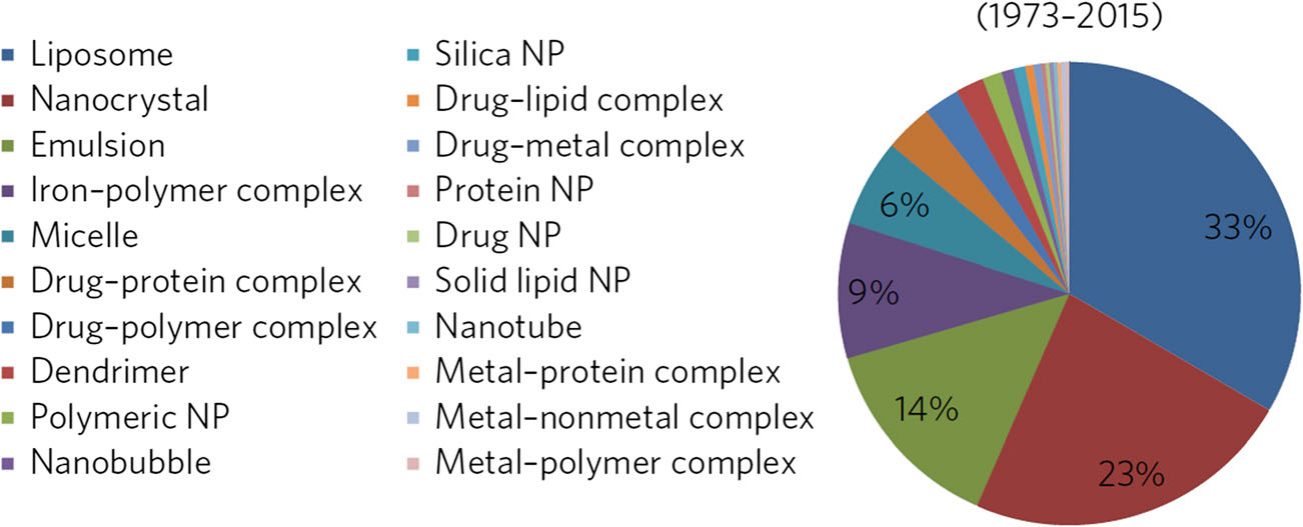
Distribution of nanomaterials use in drug products from 1973 to 2015. Breakdown of the types of nanomaterials used in drug products. The nanotechnology terminologies do not represent any implication for CDER drug product labeling and were used only to describe/interpret the type of nanomaterials in identified drug products for the purpose of this analysis (adapted from 4)

The guidance reiterates FDA’s position that it does not establish regulatory definitions for the term “nanomaterial” as explained in the guidance for industry “Considering whether an FDA-regulated product involves the application of nanotechnology” (3). Although, when looking into the history of submissions to the FDA of drug products containing nanomaterials, the following spectrum emerges (Fig. 1). This gives an idea of what FDA views—in practice—as drug products containing nanomaterials. (Draft) guidance documents for NDA and/or ANDA for a number of product classes and specific products of these listed nanomaterials were already published. A center-level guidance, however, was missing. The new guidance document, the topic of discussion for this workshop, now fills this gap.

## POSITIONING AND PREMISES OF THE NEW DRAFT GUIDANCE DOCUMENT

A common denominator for these nanomaterials is their complexity. Two themes are prominent in the guidance: (1) characterization of the nanomaterial and (2) understanding how nanomaterial attributes relate to product quality, safety, and efficacy. A risk-based approach focused on a number of listed risk factors is proposed. Debates were held on the validity and practical meaning of the listed risk factors in the discussions during the workshop. For example, can one always fully characterize these complex structures, do we always know the mechanism by which the physico-chemical properties of the material impact its biological effect, or can *in vitro* release methods indeed predict *in vivo* release?

**Fig. 2:**
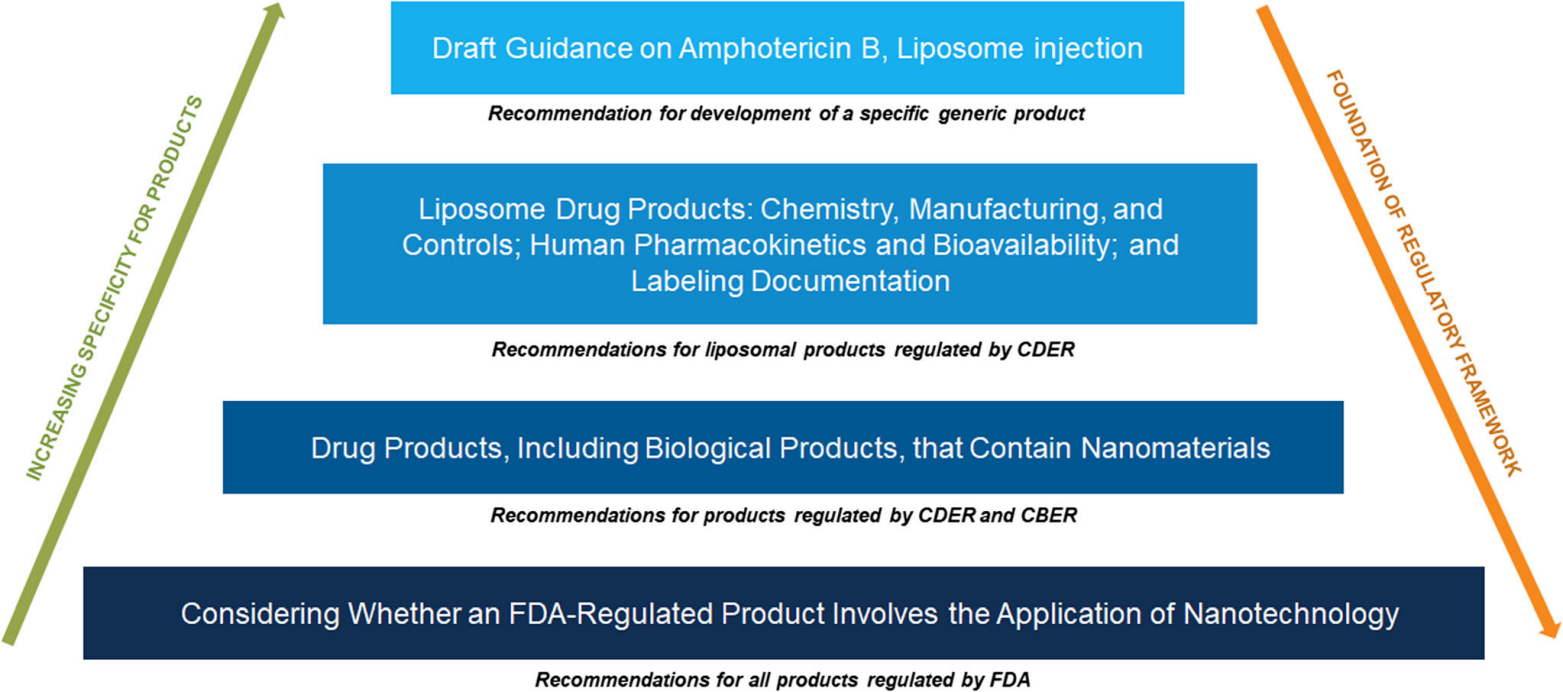
Example on how FDA guidances on the same general subject relate to each other

During the discussion, a question came up regarding how the new center-level guidance relates to older, specific nanomaterial class, or specific product-related guidances. Which guidance takes precedence? Some terms and definitions are not used in the older guidances, while they are part of the premises mentioned in the new guidance document. These older guidances may not be written with a “risk-based” approach in mind. In Fig. 2, the relationship between various nanotechnology-related guidances is explained. The FDA guidance lays the foundation of the regulatory framework for all FDA-regulated products. The CDER and CBER guidance then further provides the foundation for drug products, with class specific and product specific guidances having increasingly specific recommendations (Fig. 2).

In general, FDA guidance documents are updated to reflect current science and policy. However, the audience indicated that in some cases discrepancies were thought to exist between new and earlier issued guidance documents. This situation leads to questions for product developers.

## WHAT SHOULD DEVELOPERS TAKE AWAY FROM THIS DRAFT GUIDANCE REGARDING SUBMISSIONS OF DRUG PRODUCTS CONTAINING NANOMATERIALS?

The guidance covers a large number of topics. Its position in the “pyramid” (Fig. 2) means that this is the basic guidance for a number of structurally quite different nanomedicine subgroups and therefore does not provide product-subgroup-related detailed instructions. In the limited time available during the meeting, a selection of topics from the guidance was highlighted by Dr. Daryl Drummond, Merrimack Pharmaceuticals, based on his experience developing nanomaterials for industry, more specifically, novel liposome products. The section identifiers noted in parentheses below refer to the corresponding section in the guidance where this topic is discussed.

### Dissolution/*In Vitro* Release Testing Assays (Section IV. D)

The nanomaterial guidance says: “In general, the dissolution/*in vitro* release testing should be conducted with the drug products manufactured under target conditions and compared to drug products that are intentionally manufactured with meaningful variations in formulation and manufacturing parameters, such as particle size, drug loading, types and/or amounts of inactive ingredients.”

#### Meaningful Variations

This term is often the subject of significant debate and would benefit from greater clarification in the guidance. Although challenging—if not impossible—to establish robust *in vitro-in vivo* correlations with these assays, *in vitro* release tests still represent a promising tool for evaluating the ability to stabilize and release the drug *in vivo*, and thus distinguish poorly performing lots.

### Stability (Section IV. G)

In reference to stability, the guidance states: “The study of the stability of nanomaterials in products should involve the evaluation of physical and chemical changes in the material during handling and storage.” Dr. Drummond offered an insightful perspective into forecasting and assessing nanoparticle stability: (1) anticipate unique chemistry that results from incorporation of the active drug into a nanoparticle and recognize the impact that may have on the properties of the nanoparticle, as well as the active drug; (2) understand your process and how process-specific impacts can affect your stability; and, (3) use orthogonal methods to capture full stability impact.

### Stability upon Dilution (Section IV.G)

Additionally, the guidance reads: “In-use stability studies at clinically relevant concentrations and under relevant storage conditions may also be requested.” Information on stability upon dilution under real life conditions is indeed very relevant. One needs to consider the concentrations not only of the final drug product but also those that occur when administering the marketed drug product to the patient or concentrations needed in phase 1 trials, i.e., at much lower doses. This results in challenges in the development process. Discussions highlighted the importance of capturing this information early. Consultation with a clinician can provide insight into how the product will likely be administered, e.g., diluted in an infusion bag. This information can greatly inform the design of assays with sufficient sensitivity capable of capturing nanomaterial properties at these lower concentrations.

### Bioanalytical Methods (Section VI.D)

The paragraph describing bioanalytical methods for clinical development reads: “All clinically relevant entities, i.e., parent drug and major active metabolites, if possible, should be measured in the appropriate biologic matrices after administration of products containing nanomaterials. In general, total, free, and nanomaterial-associated drug should be measured separately or indirectly derived. This may require separation of free and nanomaterial-associated drug prior to detection or simultaneous analysis. The concentrations of free parent drug and major active metabolite(s) may be low.” Ambardekar and Stern (5) reviewed some of the most employed bioanalytical approaches to measuring drug release and discussed the pros and cons of each. Indeed, collecting accurate and precise blood/serum levels with validated assays is considered by all experts to be a significant challenge as evidenced by the abundant literature on this subject as reviewed in reference 5. This is exacerbated by the particular challenge of developing methods capable of separating very low concentrations of free drug from significantly higher quantities of nanomaterial- and protein-associated drug.

### ANDA Applications and Nanomaterials (Section VI.B)

With regard to regulatory submission, the draft guidance (1), section VI.B lines 809 onwards states: “An ANDA applicant must demonstrate, among other things, that the generic drug product is bioequivalent to the reference listed drug (RLD) (section 505(j)(2)(A)(iv) of the Federal Food, Drug, and Cosmetic Act (FD&C Act)). In addition, an ANDA must contain sufficient information to show that the proposed generic drug has the same active ingredient(s), previously approved conditions of use, route of administration, dosage form, strength, and (with certain exceptions) labeling as the RLD (section 505(j)(2)(A) and (j)(4) of the FD&C Act).” Bioequivalence studies of drug products that contain nanomaterials may be less straightforward due to the recommendation for the measurement of free and encapsulated/total forms of the drug upon administration (see above). Forum discussions highlighted that the choice of the reference listed drug product can give rise to significant challenges under circumstances of drug shortages (e.g., pegylated liposomal doxorubicin). For example, when Doxil (the reference listed drug for pegylated doxorubicin liposomes) was not available because of manufacturing problems, the generic version from Sun Pharma as approved through an ANDA was designated as reference standard. In the absence of the RLD, the reference standard can be used as the reference product for bioequivalence assessment to aid generic drug development (6). Product-specific guidances, such as the one for pegylated liposomal doxorubicin, are helpful in understanding the full scope of recommendations for equivalence of the RLD and generic product. For products without a product-specific guidance, however, it may be more difficult to understand what is needed to determine equivalence of the RLD and the generic product.

## ADDITIONAL THOUGHTS ABOUT ANDA OF NANOTECHNOLOGY PRODUCTS (ALSO REFERRED TO AS NANOMEDICINES IN THIS REPORT)

Dr. Wenlei Jiang from the FDA (Office of Research and Standards, Office of Generic Drugs, Center for Drug Evaluation and Research) gave her personal views on regulatory research in nanomedicine with a focus on generics. FDA recognizes the complexity of nanotechnology products and strongly encourages regulatory research in this area. FDA has a strong program in place to support nanotechnology research and CDER’s nanotechnology research has focused on quality, safety, equivalence, and post-market surveillance of these products via internal projects and extramural grants/contracts. The outcomes of various studies become scientific bases for FDA general guidances and product-specific guidances for nanotechnology drug products, which are made available in the public domain (7,8). Results from industrial and academic studies related to these regulatory topics are also welcome.

**Fig. 3:**
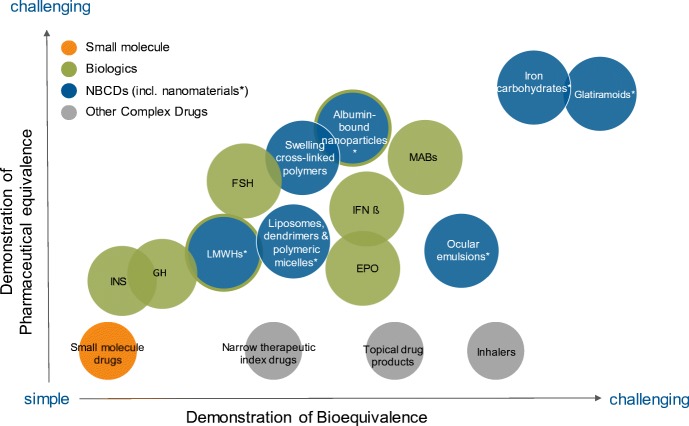
The complex drug landscape (illustrative). Drug products are positioned on the basis of the challenge to assess pharmaceutical equivalence (PE) and bioequivalence (BE) of two drug products (i.e., the reference product and its generic version). Conventional low-molecular-weight drugs that can be fully characterized are shown in orange; demonstration of PE and BE is relatively simple. Biologics are shown in green, NBCDs are shown in blue, and other complex drugs are shown in gray. Albumin-bound nanoparticles and low-molecular-weight heparins are blue with a green outline (classification of these drugs varies across the globe). Adapted from Hussaarts *et al.* (10)

During interactions with the audience, the point was made that establishing TE (therapeutic equivalence) for a generic nanotechnology product may be a challenge. When using the generic drug approval paradigm, the generic product should be PE (pharmaceutically equivalent) and BE (bioequivalent). Establishing PE for some nanotechnology products may be difficult. And although FDA does not adopt the term non-biological complex drugs (NBCDs), several of these products of which many are nanomaterial drug products face the same challenges as biologics in the determination of PE and BE (9), Fig. 3. For NBCDs, it has been documented that “the process is the product,” i.e., a well-controlled and well-understood manufacturing process should be in place to ensure reproducible product quality (11–13). As with other drug products, depending on drug toxicity, BE studies of nanomedicines can be conducted either in healthy subjects or in patients (14,15). Alternative options may encompass establishing equivalence using (pharmacodynamic) biomarkers or clinical endpoints. The applicant for an ANDA for a nanomedicine through the 505(j) pathway may need guidance on how to establish TE. To address this issue, the FDA is adopting a case-by-case approach and providing recommendations on how to demonstrate PE and BE per nanomedicine product class or for a specific drug product.

For parenteral nanoparticle dosage forms, the FDA recommends the Q1 and Q2 of the generic product and RLD to be same.Q1 and Q2 refer to the qualitative and quantitative composition, respectively, of the inactive ingredients compared with the RLD. Besides Q1 and Q2 sameness, physico-chemical sameness/equivalence of parenteral nanoparticles, e.g., particle size distribution, morphology, and lamellarity, are recommended to ensure the high-order structure sameness. The draft guidance states that “the active ingredients of some nanomaterials are generally heterogeneous mixtures which may require considerable characterization to demonstrate drug substance sameness.” Also, the guidance highlights that it is critical to identify the most therapeutically relevant moiety for establishing BE. Furthermore, drug levels in systemic circulation may not always reflect drug concentration at the target site. As a result, in most cases, evidence of comparable pharmacokinetic (PK) parameters in blood/plasma in conventional BE studies alone may not be sufficient to satisfy the requirements for generic drug approval. For complex dermatological preparations like creams and emulsions, assessment of a similar microstructure arrangement of the matter is also recommended. The question was raised why the term Q3 is not used for generic nanomaterial products as the FDA requests—de facto—a higher level of comparison to establish PE by examining the arrangement of matter, i.e., the microstructure. The Q3 term, as highlighted by regulatory scientists present in the meeting, is never an official term in any guidance document. Instead, *in vitro* characterization or *in vitro* physico-chemical characterization is used.

The NDA 505(b)(2) route may be used for products closely related to a reference product, and section VI a of the draft guidance describes how FDA categorizes different products following a risk-based approach. Depending on the evidence present and the purpose of the development program, the extent of potential clinical development may vary from comparable plasma PK bioequivalence to comparative safety and efficacy studies. In some cases, non-inferiority and/or superiority study designs can be considered. Important to note, approval through 505(b)(2) will, per definition, not lead to a generic product since it follows the NDA route, leading to a product that cannot be substituted with the reference product.

Breakout session facilitators included Dr. Jon de Vlieger, Dr. Scott McNeil, Dr. Sesha Neervannan, and Dr. Daryl Drummond. In the breakout sessions, the two questions raised by the AAPS membership, as well as a number of other related issues, were discussed. The section below describes the discussions, reflections, and outlook, partly summarized as wrap up of the meeting by Dr. Daan Crommelin.

## CQA ASSESSMENT FOR COMPLEX DRUG PRODUCTS

In the guidance, CQA assessment is a major pillar for structuring the risk-based analysis. For example, it reads: “The CQAs need not be an exhaustive catalogue of quality attributes, but should capture attributes that potentially impact the quality, safety, or efficacy of the final product.” From an industrial perspective, certain well-established nanomaterial characteristics that are known to impact performance (e.g., clearance rates), such as particle size or surface charge, are provided for specifically in the guidance. However, many are product specific. Furthermore, it is beneficial to create a table at the onset of development for any new product candidate, listing the characteristic, the mechanism behind its theoretical impact on product performance, and the initial specifications. This should be updated during development as more data becomes available around the impact of ranges of characteristic parameters, or upon the identification of new attributes. Determining CQA’s and their related specifications is beyond a theoretical exercise and benefits from experimental data to establish the impact of meaningful changes to the CQAs, notably at the boundary of proposed specifications. Ultimately, the impact on clinical performance validates the approach taken to identify the attributes.

The question was brought up: who identifies CQAs and defines the design space? In terms of defining the design space, generic companies have a clearly defined window to work with as set by the relevant quality parameters of the originator product. The answer to “who identifies CQAs?” is that each developer, for an innovator or generic product, is responsible for noting the CQAs for their product. Obviously, innovator companies do not disclose them as they constitute confidential information. And, FDA cannot disclose these either. If a generic company develops a CQA profile for a generic product, it is likely to be challenged by the innovator company. What happens when the CQAs identified by the innovator and generic developer differ? How will FDA respond? This catch-22 situation was discussed at the breakout session, and although a case-by-case approach was proposed, a conclusion on potential structural solutions was not reached.

### Hard Data, Please

A number of publications clearly describe the strategy to identify CQAs. However, many of these publications refrain from making statements on the quantitative parameters for the specific CQAs (16–19). For complex nanomedicine drug products, only one publication could be found on CQA assessment, including such specification of ranges. Troiano *et al.* describe how they used a quality by design approach for developing nanomedicines. More specifically, by using a risk-based approach to identify and classify product attributes and process parameters, they ultimately developed a deep understanding of the products, processes, and platform (20). Although the focus of the approach is on CMC affairs, significant consideration is also given to preclinical, clinical, and regulatory aspects of pharmaceutical development. Following this exemplary paper, more publications with hard data on this issue for other technology platforms or products are highly desired. Dr. Jiang pointed out that, in the context of the GDUFA (Generic Drug User Fee Amendments) funded research on nanotechnology products, FDA is committed to performing research (internal and extramural) on topics related to quality assessment for ANDAs for complex drugs such as iron-gluconate and several issues related to liposome characterization. The outcome of these FDA-funded research projects will be shared with the scientific community through publications and will be open for discussion. In addition to publishing the outcomes of FDA funded research, it was suggested that FDA could publish some blinded case studies to support enriching the scientific base for nanotechnology products. In the discussion during the workshop, industry scientists were urged to publish their experience on the assessment of CQA and design space in the public domain as well.

### CQA Assessment for Biologics: a Role Model

Although excluded from the draft guidance discussed during the meeting, biologics are (highly) complex drugs as well and one may learn from the experience obtained over the last decade. Numerous informative publications are available for biologics giving examples of real-data assessment of CQAs and their design space (21–23). After a series of inter-company and regulatory interactions, the CMC Biotech Working Group, consisting of experts from seven biotech product developers, published an extensive case study on therapeutic monoclonal antibody bioprocess development (22). This document, counting over 270 pages, is by far the closest attempt to come to an exhaustive catalog of CQAs published. For biosimilars, Vandekerckhoven *et al.* described scientific and methodological considerations on the process of attribute and test method selection, criticality assessment, and subsequent assignment of analytical measures to U.S. FDA’s three tiers of analytical similarity assessment (23). These approaches taken to determine and specify the CQAs for biologics could inspire developers of complex nanomedicine drug products to do the same.

## A QUESTION OF THE REGULATORY PATHWAY FOR NANOMEDICINES: A SIMILAR PATHWAY FOR NANOSIMILARS AS FOR BIOSIMILARS?

During the meeting and following the paper by Marden *et al.* (24), some participants made the point that the 505(j) route may not be appropriate in the case of drug products containing nanomaterials. Terminology-wise, the wording “complex generic” implies therapeutic equivalence and thus substitutability. In terms of complexity, complex nanomedicine products show resemblance to biologic products and therefore it was proposed to discuss a potential new regulatory pathway for nanomaterials that would be like the biosimilar pathway. The FDA staff participating in the meeting raised strong concerns that this topic was out of scope for a workshop intended to focus on the scientific issues related to nanomedicines.

Meeting attendees did agree that (i) Some of the nanomaterials are difficult to be fully characterized leading to challenges in demonstrating pharmaceutical equivalence (PE challenges); (ii) A good understanding and proper control over the manufacturing process is critical for a reproducible, high-quality drug product; (iii) CQA assessment protocols are requested and a risk-based approach is recommended; and (iv) there may be questions related to bioequivalence (BE) testing as bioequivalence assessment is often more challenging than for conventional formulations. It is agreed that robust analytical studies and a stepwise approach in comparing the generic product and reference product will provide the necessary information leading to a decision on therapeutic equivalence of these products. Recognizing the complexity in establishing PE and BE, the FDA staff indicated they will continue efforts in developing product-specific guidances to guide generic nanotechnology drug product development.

## HARMONIZATION: DIVERGING STANDARDS?

Over time, differences in approval standards between different regions in the world have been observed, leading to additional efforts and increased costs (e.g., extra patient involvement) for both the innovative and generic industry. Examples are the different approval processes for complex generic drug products in the USA and the EU for LMWH (low molecular weight heparins), glatiramoids, iron-carbohydrate products, and doxorubicin HCl liposomes. Harmonization is critical for developers. When multiple new questions arise regarding nanomedicine development protocols in each and every new market, the approval of these much-needed candidate nanomedicines in these markets can be significantly delayed or even discontinued. Although not discussed in detail, the question was raised whether it is time for an ICH initiative, or to step up other initiatives to harmonize (parts of) the standards for approval of nanomedicines. During time of writing of this report, a statement of the FDA Commissioner Gottlieb was published in support of global harmonization of scientific and technical requirements for generic drugs. The ultimate goal of this global harmonization, as written in the statement, would be the attainment of a single global generic drug development program that can support simultaneous regulatory filings across multiple markets (25).

## CONCLUSION—NEXT STEPS?

The AAPS Guidance Forum on the draft guidance, “Drug Products, Including Biological Products, that Contain Nanomaterials” proved to serve as an open discussion platform for scientists from industry, academia, and regulatory bodies. Such an open discussion provides relevant input for industry and regulatory authorities on how draft guidances are (to be) interpreted. For the particular draft guidance at hand, the formal commenting period was already closed at the time of the meeting. However, speakers from FDA invited participants to bring any questions to their attention to avoid any doubt on how to read and interpret guidances for the development of NDAs and ANDAs. Although aware of short timelines, the participants agreed that alignment of the release of guidance documents and the organization of public discussions such as in the AAPS Guidance Forum is of mutual benefit to both regulators and industry stakeholders. This would allow for in-depth, stakeholder-broad discussions of draft guidance documents, facilitating drug development and regulatory approval process for safe and effective nanomedicines—new or generic.
